# A path analysis of the effects of the doctor-patient encounter and expectancy in an open-label randomized trial of spinal manipulation for the care of low back pain

**DOI:** 10.1186/1472-6882-14-16

**Published:** 2014-01-13

**Authors:** Mitchell Haas, Darcy Vavrek, Moni B Neradilek, Nayak Polissar

**Affiliations:** 1 Center for Outcomes Studies, University of Western States, 2900 NE 132nd Ave., Portland, OR, USA; 2 The Mountain-Whisper-Light Statistics, 1827 23rd Ave. East, Seattle, WA, USA

**Keywords:** Statistical model, Chronic low back pain, Spinal manipulation, Chiropractic, Randomized controlled trial, Doctor-patient interaction, Expectations

## Abstract

**Background:**

The doctor-patient encounter (DPE) and associated patient expectations are potential confounders in open-label randomized trials of treatment efficacy. It is therefore important to evaluate the effects of the DPE on study outcomes.

**Methods:**

Four hundred participants with chronic low back pain (LBP) were randomized to four dose groups: 0, 6, 12, or 18 sessions of spinal manipulation from a chiropractor. Participants were treated three times per week for six weeks. They received light massage control at visits when manipulation was not scheduled. Treating chiropractors were instructed to have equal enthusiasm for both interventions. A path analysis was conducted to determine the effects of dose, patient expectations of treatment success, and DPE on LBP intensity (100-point scale) at the end of care (6 weeks) and primary endpoint (12 weeks). Direct, indirect, and total standardized effects (β_total_) were computed. Expectations and DPE were evaluated on Likert scales. The DPE was assessed as patient-rated perception of chiropractor enthusiasm, confidence, comfort with care, and time spent.

**Results:**

The DPE was successfully balanced across groups, as were baseline expectations. The principal finding was that the magnitude of the effects of DPE on LBP at 6 and 12 weeks (|β|_total_ = 0.22 and 0.15, p < .05) were comparable to the effects of dose of manipulation at those times (|β|_total_ = 0.11 and 0.12, p < .05). In addition, baseline expectations had no notable effect on follow-up LBP. Subsequent expectations were affected by LBP, DPE, and dose (p < .05).

**Conclusions:**

The DPE can have a relatively important effect on outcomes in open-label randomized trials of treatment efficacy. Therefore, attempts should be made to balance the DPE across treatment groups and report degree of success in study publications. We balanced the DPE across groups with minimal training of treatment providers.

**Trial registration:**

ClinicalTrials.gov NCT00376350

## Background

The therapeutic effects of care are complex and not limited to the consequences of a specific intervention [1]. Hence, nonspecific effects of care clearly need to be addressed in the design of randomized trials. Patient response expectancy can be an important contributor to the treatment effect [2,3]. Provider enthusiasm during the treatment visit can also have a positive effect on outcomes [4].

Four trials and a nonrandomized comparison study of care for low back pain (LBP) have reported the effects of baseline expectation of treatment success on functional disability with mixed results. For acute LBP, general expectancy of success, but not expectation of specific treatments’ success, was observed to be a determinant in a choice trial that included chiropractic as an option [5]. The nonrandomized study found important and comparable effects of expectation for both manual and mechanical spinal manipulation techniques by chiropractors [6]. For chronic LBP, expectation of success in the treatment that the participant received was an important determinant of outcomes in a comparison of acupuncture and massage [7]. Expectancy also affected outcomes in a trial comparing physical therapy, cognitive-behavioral therapy and combination of the two [8]. In contrast, expectation of success did not affect outcomes in a more recent trial comparing acupuncture, sham acupuncture, and medical care [9]. For pain outcomes, the nonrandomized study on acute LBP found no effect of expectation, in juxtaposition to the effect on functional disability [6]. None of these studies looked at the influence of evolving expectations over time, as well as the effect of doctor-patient encounter (DPE) on outcomes.

Blinding of participant and provider are important for establishing the internal validity for randomized trials of treatment efficacy [10] and are universally included as important components of quality scoring instruments used for systematic reviews of randomized trials [11,12]. Blinding serves to control the effects of expectancy of treatment success and the nonspecific effects of DPE across groups in studies of efficacy, where the goal is to isolate the specific effects of an intervention above and beyond its social contexts. Unfortunately, it is often not possible to blind patients and providers in studies evaluating the efficacy or relative efficacy of manual therapies.

With this in mind, we took steps in an earlier dose–response trial on spinal manipulation for cervicogenic headache to help minimize the effects of expectation and the doctor-patient encounter (DPE) [13,14]. Chiropractors were asked to interact with the study participants with uniform enthusiasm across study groups. The chiropractors were monitored by participant questionnaire over time and clinical observation. The questionnaires revealed that the providers could indeed interact with patients receiving different therapies with equipoise. Furthermore, expectations over time and the DPE had little effect on pain outcomes compared to treatment intervention and baseline pain [14].

These steps were repeated for the current study. We conducted a much larger randomized trial evaluating the dose–response and efficacy of spinal manipulative therapy provided by a chiropractor for the care of chronic LBP [15]. We found that the dose–response gradient was small and treatment had a modest advantage over control. The purpose of this report was to confirm the ability to balance the DPE over treatment groups, and to present a path analysis modeling the effects of ongoing expectation of success and DPE on chronic low back pain outcomes.

## Methods

### Design

The study is presented in detail in the principal report of outcomes [15]. In a prospective open-label, randomized controlled trial, 400 participants with chronic LBP were randomized to receive a dose of 0, 6, 12, or 18 sessions of SMT from a chiropractor. The protocol called for all participants to attend 18 visits, three per week for six weeks. At non-SMT visits, a brief, light massage control was performed to control provider attention and touch by the chiropractor. That is, the number of control visits equaled 18 minus the number of SMT visits. The study was conducted in the Portland, Oregon metropolitan area between March 2007 and July 2011.

Computer generated design adaptive allocation [16,17], implemented immediately before the first treatment, was used to conceal assignment prior to randomization and balance important variables at baseline. These included age and gender, as well as variables relevant to controlling outcomes and participant expectations: pain and disability scores, previous care from an SMT or massage provider, and the relative confidence of the participant in the success of SMT and massage care for chronic LBP. Data for this report were collected at baseline and 12-wk primary endpoint by written questionnaire and by blinded phone interview two to seven days following the last treatment at 6 weeks.

### Protocol

Participants were recruited through craigslist, mailers, and local newspapers. They were informed during a scripted interview that the study was investigating 18 visits for different combinations of two manual therapies for chronic LBP: SMT and light massage. They were told dispassionately that there was potential benefit from either intervention alone or in combination. Eligibility screening examinations were conducted at a central university clinic by one of two chiropractors using history, physical exam, and lumbar x-rays [18]; they gave the participants detailed descriptions of the SMT and massage protocols. Eligible participants selected a convenient clinic for study care. Care was provided by one of 12 licensed chiropractors with 4 to 24 years of experience in nine Portland-area clinics. Participants were compensated for each treatment visit, mailed questionnaires, and phone interviews ($10 to $20). Participants signed an informed consent form. Protection of human subjects was approved by the University of Western States Institutional Review Board.

The treating chiropractors interacted with the patients at each 15-minute treatment visit. They took brief histories and chatted with the patients as in usual practice. Of particular pertinence to this report, the chiropractors were asked to interact with the participants with equal enthusiasm for care across the different interventions and doses of care. This was instituted to help create balance of expectations for treatment success imparted by the practitioner.

The need for equipoise was discussed during two training sessions in the preparation phase of the study and reinforced during quarterly training sessions. A study investigator (chiropractor) also gave feedback to the treating chiropractors if he noted any patterns of unintentional breach of equipoise across groups during study visit observations. Equipoise in enthusiasm and other variables were evaluated through patient questionnaire described below [13-15].

### Participants

Participants were required to be at least 18 years old, have a current episode of chronic LBP [19] of mechanical origin [20] of at least three months duration [21], have had some LBP on 30 days in the prior six weeks, and had a minimum LBP index of 25 on a 100-point scale to prevent floor effects. Participants were excluded if they received manual therapy within the previous 90 days. They were also ineligible if they had contraindications to study interventions [18,22] or complicating conditions such as active cancer, spine pathology, inflammatory arthropathies, autoimmune disorders, anti-coagulant conditions, neurodegenerative diseases, pain radiating below the knee, organic referred pain, pregnancy, and disability compensation.

### Intervention

Participants spent 15 minutes with a treating chiropractor. They received a hot pack for five minutes to relax spinal muscles followed by five minutes for the SMT or control intervention. The visit was completed with five minutes of very low dose pulsed ultrasound (20% duty cycle with 0.5 watts/cm^2^). This was used as a quasi-sham to enhance treatment credibility and adherence to care [23].

SMT consisted of thrust spinal manipulation in the lumbar and transition thoracic regions, predominantly in the side-posture position [24]. Specific manipulations to be performed were determined at each visit by the chiropractor through ongoing evaluation of the participants [18]. The light massage control consisted of five minutes of gentle effleurage and petrissage of the low back (lumbar and lower thoracic) paraspinal muscles [24,25], focused on the symptomatic areas. The massage used was gentler and of shorter duration than recommended for therapeutic massage practice [26,27]. It was a minimalist intervention to control touching the patient and was not a formal sham.

### Study variables in the analysis

Low back pain intensity, a primary study outcome, was evaluated using the Modified Von Korff pain scale of Underwood et al. [28]. It is the average of three 11-point numerical rating scales pain today, worst pain in the last 4 wk, and average pain in the last four wk. The scale is scored from 0 to 100 with a lower score being more favorable. The scale has been shown to be reliable, valid, and responsive for measuring pain (including headache) and was chosen for its brevity, simplicity, acceptability to participants, and validity as a phone questionnaire [28].

Patient expectation was evaluated as confidence in the success of assigned study intervention using Interstudy’s Low Back Pain TyPE Specification instrument [29]. The 6-point Likert scales were anchored by “extremely doubtful” and “extremely certain”. Expectation of success for spinal manipulation and light massage were evaluated at baseline prior to randomization. The specific questions were: “How confident are you that 18 visits for spinal manipulation plus ultrasound therapy will be able to successfully treat your back problem?” and “How confident are you that 18 visits for light massage plus ultrasound therapy will be able to successfully treat your back problem?” The average value of the two scales was included in the analysis. At the 6- and 12-wk follow-ups, a single question was administered: “How confident are you that the care you received is working?”

Measures of the DPE included patient perception of chiropractor enthusiasm for care, comfort treating low back pain, confidence in care success, and adequate time spent with the patient. An example question was, “My doctor seemed enthusiastic about my treatment program”. The four variables were measured on 5-point Likert scales anchored by “strongly disagree” to strongly agree” [30,31]. A “could not tell” response option was added for the enthusiasm, comfort, and confidence questions based on participant feedback in our previous study [14]. This response was coded as the neutral score 3 on the 5-point scale. The average of the four scales was used to create a composite DPE measure for the analysis.

### Statistical analysis

Path analysis fitted using structural equation modeling software [32,33] was conducted to identify the direct, indirect, and total effects of manipulation dose, prior low back pain intensity the DPE, and patient expectancy of treatment success on chronic LBP intensity. This was a preplanned secondary analysis of our study data.

● The direct effect (β_direct_) of one variable on another variable in the model is described by a path coefficient. The coefficient represents the effect of an independent variable (v1) on a dependent variable (v2) controlling for other predictors of v2 in the model, as in a multiple regression analysis. The standardized path coefficients correspond to all variables in the analysis standardized to a mean of zero and a variance of 1.0. This standardization yields path coefficients on a common scale (from −1 to 1) and enables comparison of effects of different variables. Quantitatively, the standardized path coefficient represents the change in the dependent variable in standard deviations for a one standard deviation change in the independent variable (larger absolute values of the coefficient correspond to stronger effects). The coefficients are included in the graphical depiction of the model. The arrows represent the direction of presumed influence (v1 → v2).

● The indirect effect (β_indirect_) is the effect of independent variable (v1) on dependent variable (v2) that is mediated by variables on the paths between v1 and v2. For example, assume that v2 has six predictors (v1 to v6) and that the indirect effect contains three paths with mediating variables v3 and v4 (v1 → v3 →v2, v1 → v4 →v2, and v1 → v3 → v4 →v2). The indirect effect is quantified by first computing the product of the path coefficients on each of the three paths and then summing the products for the three paths. The indirect effect is controlled for all predictors of v2 not serving as mediating variables on the paths, in this case v1, v5, and v6.

● The total effect (β_total_) of one variable on another is the sum of the direct and indirect effect connecting the two variables: β_total_ = β_direct_ + β_indirect_. The total effect represents the effect of v1 on v2 that is independent of all variables that are, according to the model, not caused (directly or indirectly) by v1. The total effect is important because it expresses the total estimated effect of v1 on v2. Note that the total effect can be meaningfully larger or smaller than the direct effect (path coefficient).

Modeling was guided by several principles in determining potential pathways of influence of one variable on another or, stated simply, the presence and direction of pathway arrows between variables in the path analysis model diagram. 1) Both SMT dose and DPE were considered to be interventions. 2) Dose was assumed to affect DPE and expectations. 3) Baseline pain was assumed to affect pain at 6 weeks and at 12 weeks. 4) Baseline expectations were assumed to affect expectations at 6 weeks and at 12 weeks. 5) Baseline expectations were also assumed to affect the DPE at 6 weeks. 6) All variables evaluated at 6 weeks were presumed to influence all variables at 12 weeks. 7) DPE was assumed to affect concurrent pain and expectations and pain was assumed to affect concurrent expectations. 8) It was noted that feedback loops, such as pain influencing expectations and expectations influencing pain, could not be incorporated in the models.

Models were fit by the maximum likelihood method. Confidence intervals and p-values were calculated by the bootstrap with 500 resamples of patients with replacement. The fitted model was considered satisfactory because all of the following conditions were met (model fit in parentheses): a) a statistically non-significant likelihood ratio test of the fitted model vs. the saturated model, indicating no important paths were omitted (model: p = .11); b) a statistically significant likelihood ratio test of the model vs. the baseline model, indicating improvement against a model that assumes no non-null associations (model: p < 0.001); c) the root mean squared error of approximation RMSEA <0.05, indicating small residual variation (model: 0.043); and d) the comparative fit index CFI > 0.95 (model: 0.996) and the Tucker-Lewis index TLI > 0.95 (model: 0.997), supporting a favorable comparison of our model to the saturated and baseline models. The multivariate normality assumption was confirmed by normal quantile-quantile plots of residuals. Strong multicollinearity was ruled out using the variance-inflation factor.

Treatment was included as a dose variable representing the linear effect of the number of visits for spinal manipulation (0, 6, 12, or 18.) In a second analysis, three grouping variables were introduced to evaluate the effects of the three manipulation dose groups compared to the control on the model outcomes.

Each participant was included in the original allocation group (intention-to-treat analysis). Missing data were imputed using linear interpolation, when bracketing data were available, and last datum carried forward when last data points were unavailable [15]. Nine participants were omitted from the analysis because they had no follow-up data, so that 391 were included in the analysis. The sample size was set in advance to have 80% power to detect a 10-point difference between two treatment groups on the 100-point pain scale using a 2-sided test at the .025 level of significance [15]. For the path analysis, statistical significance was set at the .05 level. All analyses were conducted with Stata 11.2 (Stata Corp, College Station, TX).

## Results

**Figure 1:**
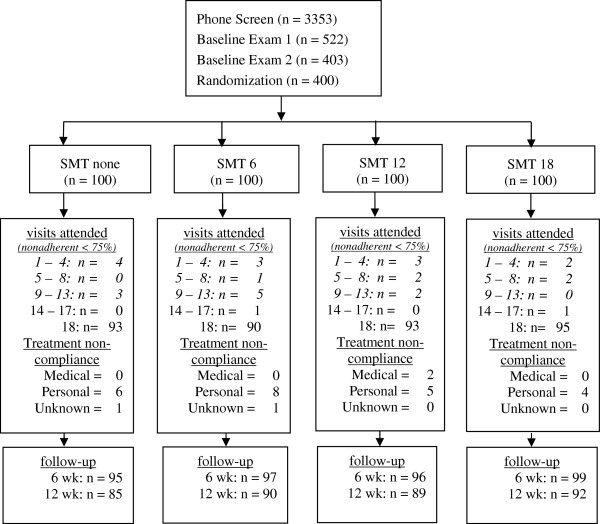
Study flowchart.

Figure 1 shows adherence to care and compliance with follow-up. Further details are published elsewhere [15]. There was strong adherence to care with 90% to 95% of participants in each group attending all 18 study visits. Compliance with follow-up data collection was also very good: 95% to 99% at six weeks and 85% to 92% at 12 wk. During the treatment phase, 93% to 97% of participants in each treatment arm refrained from professional care outside of the study.

The mean participant age was 41.3 years and 85% were white non-Hispanic. Approximately half reported each of the following characteristics: female, college degree, comorbidity, and experience with a study intervention. The mean duration of low back pain was 11.8 years and the mean frequency was 6 days per wk. Baseline characteristics were well balanced across groups [15].

### Outcomes

Variables included in the path analysis are shown in Table 1. Mean pain improvement for each group at both follow-ups is considered clinically important for an individual patient [34-36]. Mean improvement at the end of care was durable to 12 weeks.

**Table 1:** Variables in the path analysis models*

**Week**		**n**	**0 SMT**	**n**	**6 SMT**	**n**	**12 SMT**	**n**	**18 SMT**	**n**	**All**
Low back pain											
Pain intensity (0 – 100: mean of pain today, worst pain in last 4 weeks, and average pain over last four weeks)											
0		95	52.2 (16.3)	99	51.0 (18.2)	97	51.6 (17.5)	100	51.5 (16.8)	391	51.6 (17.2)
6		95	34.5 (18.4)	99	32.3 (15.8)	97	27.1 (14.7)	100	30.2 (19.0)	391	31.0 (17.2)
12		95	37.9 (20.4)	99	32.7 (19.4)	97	29.0 (20.8)	100	31.4 (19.8)	391	32.7 (20.3)
Expectations (6-point Likert scale: 0 extremely doubtful, 3 unsure, and 6 extremely certain)											
Confidence at baseline											
0	in SMT	95	3.6 (1.2)	99	3.8 (1.1)	97	3.7 (1.2)	100	3.8 (1.1)	391	3.7 (1.2)
0	in LM	95	3.4 (1.2)	99	3.5 (1.2)	97	3.4 (1.2)	100	3.5 (1.2)	391	3.5 (1.2)
0	average	95	3.5 (1.1)	99	3.7 (1.1)	97	3.5 (1.1)	100	3.7 (1.1)	391	3.6 (1.1)
Confidence that care received is working											
6		95	3.2 (1.8)	97	3.9 (1.7)	96	4.4 (1.3)	99	4.2 (1.6)	387	3.9 (1.7)
12		86	3.1 (1.8)	90	3.2 (1.7)	88	3.9 (1.4)	92	3.7 (1.6)	356	3.5 (1.7)
Doctor-patient encounter (5-pt Likert scale: 1 strongly disagree to 5 strongly agree)											
My doctor seemed comfortable dealing with my back pain											
6		95	4.8 (0.5)	97	4.8 (0.7)	96	4.8 (0.5)	99	4.8 (0.4)	387	4.8 (0.5)
12		86	4.5 (0.8)	90	4.6 (0.7)	89	4.5 (0.9)	92	4.5 (0.8)	357	4.5 (0.8)
My doctor seemed enthusiastic about my treatment program											
6		95	4.4 (0.9)	97	4.4 (0.8)	96	4.6 (0.8)	99	4.5 (0.7)	387	4.5 (0.8)
12		86	4.0 (1.0)	90	4.2 (0.9)	89	4.1 (1.0)	92	4.1 (1.0)	357	4.1 (1.0)
My doctor spend adequate time listening to my description of the pain											
6		95	4.6 (0.8)	97	4.7 (0.7)	96	4.7 (0.7)	99	4.7 (0.6)	387	4.7 (0.7)
12		85	4.6 (0.8)	89	4.4 (0.9)	89	4.5 (1.0)	92	4.5 (0.8)	355	4.5 (0.9)
My doctor seemed confident that the treatment provided would work											
6		95	3.4 (0.9)	97	3.6 (0.9)	96	3.7 (0.9)	99	3.7 (0.9)	387	3.6 (0.9)
12		86	3.3 (0.9)	90	3.6 (1.0)	89	3.6 (1.0)	92	3.5 (1.0)	357	3.5 (1.0)
Average patient impression of DC comfort, enthusiasm, confidence in tx success, and adequate time listening											
6		95	4.3 (0.5)	97	4.4 (0.5)	96	4.4 (0.5)	99	4.4 (0.4)	387	4.4 (0.5)
12		85	4.1 (0.7)	89	4.2 (0.7)	89	4.2 (0.8)	92	4.1 (0.7)	355	4.2 (0.7)

*The Mean (SD) is presented for each variable in the model for each spinal manipulation dose level (0, 6, 12, and 18 manipulation visits) and the total sample. Data are presented for model variables at baseline (0 weeks), end of care (6 weeks), and subsequent follow-up (12 weeks). Note that the variables in the model for baseline expectations and the doctor-patient encounter are computed averages of preceding variables in the table.

*SMT* spinal manipulative therapy (spinal manipulation).

Confidence in SMT and light massage was successfully balanced across treatment groups at baseline by the allocation program. Overall baseline mean scores demonstrated little certitude for success of care for either SMT or light massage (3.7 and 3.5, respectively).

Pairwise comparisons to the control group showed that expectations were greater in the two highest dose groups at both follow-up time points (t-tests, p < .05). This is commensurate with the greater improvement in pain seen in the highest dose groups (Table 1) [15].

We were successful in achieving uniform patient perceptions of the doctor-patient encounter across treatment groups at both 6 and 12 weeks. Group means for the composite score differed by 0.1 or less on the 5-point scale (p > .05).

### Pairwise correlations

Pairwise correlations between variables in the model are presented in Table 2. The strongest correlations were between like variables at the different time points, such as pain at six and 12 weeks (r = 0.72). There was also substantial correlation between follow-up pain and expectations. The association of DPE with pain and expectation were not as large. Notably, dose was not strongly correlated with any variable.

**Table 2:** Correlations between model variables*

	**Lowback pain**	**Expectation**	**Dose**	**Lowback pain**	**Dr-Patient encounter**	**Expectation**	**Lowback pain**	**Expectation**	**Dr-Patient encounter**
	**Baseline**	**Baseline**		**6 weeks**	**6 weeks**	**6 weeks**	**12 weeks**	**12 weeks**	**12 weeks**
Low back pain – baseline		**0.27**	−0.01	**0.44**	0.00	0.01	**0.41**	0.00	0.00
Expectations – baseline		-	0.04	0.07	**0.20**	**0.14**	0.07	**0.15**	**0.13**
Dose		-	-	**−0.12**	**0.23**	0.06	**−0.13**	**0.17**	−0.01
Low back pain – 6 weeks		-	-	-	**−0.50**	**−0.23**	**0.72**	**−0.44**	**−0.19**
Expectations – 6 weeks		-	-	-	-	**0.35**	**−0.42**	**0.73**	**0.33**
Dr-Patient encounter – 6 weeks		-	-	-	-	-	**−0.16**	**0.30**	**0.59**
Low back pain – 12 weeks		-	-	-	-	-	-	**−0.47**	**−0.17**
Expectations – 12 weeks		-	-	-	-	-	-	-	**0.34**
Dr-Patient encounter – 12 weeks									

*Pearson’s correlation coefficient shows the univariate correlation between variables.

Bolded coefficients are statistically significant (p < .05).

### Path analysis model

**Figure 2:**
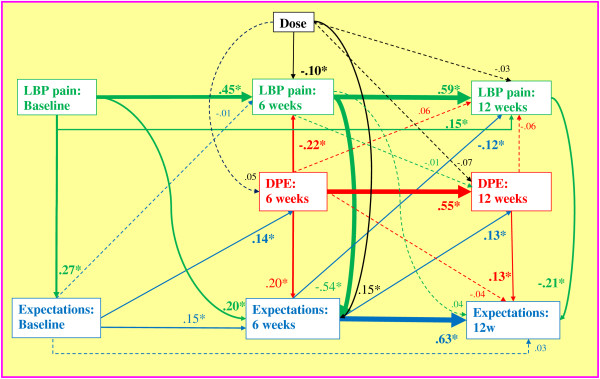
**Path analysis diagram.** Standardized path coefficients are presented. The absolute magnitude indicates the strength of the relationship between variables and the arrow points to the presumed direction of influence. Solid arrows indicate statistical significance (p < .05) and the width of solid arrows indicates the magnitude of the coefficient. Color of the arrow indicates variable of origin; green = pain, red = DPE, blue = expectations, black = treatment dose.

The path analysis schematic with the estimated direct effects is presented in Figure 2. Thicker arrows show larger direct effects. Standardized coefficients with 95% confidence intervals for direct, indirect, and total effects are included in Table 3 for all variables in the model; boldface results are statistically significant at the p < 0.05 level. Unless otherwise noted, the results reported in the remainder of the text are statistically significant and include only the total effects abbreviated as β.

**Table 3:** Path analysis*

**Dependent variables**	**Direct effects**		**Indirect effects**		**Total effects**	
**Determinants**	**β**	**95% CI**	**β**	**95% CI**	**β**	**95% CI**
Expectations – baseline (R^2^ = 0.07)						
Low back pain – baseline	**0.27**	(0.17, 0.37)			**0.27**	(0.17, 0.37)
Doctor-Patient encounter – 6 weeks (R^2^ = 0.02)						
Expectations – baseline	**0.15**	(0.04, 0.26)			**0.15**	(0.04, 0.26)
Low back pain- baseline	−0.03	(−0.14, 0.08)	**0.04**	(0.01, 0.07)	0.01	(−0.09, 0.11)
Dose	0.05	(−0.05, 0.16)			0.05	(−0.05, 0.16)
Low back pain – 6 weeks (R^2^ = 0.26)						
Expectations – baseline	−0.01	(−0.11, 0.08)	**−0.03**	(−0.06, -0.01)	−0.05	(−0.14, 0.05)
Doctor-Patient encounter – 6 weeks	**−0.22**	(−0.31, -0.13)			**−0.22**	(−0.31, -0.13)
Low back pain- baseline	**0.45**	(0.36, 0.54)	−0.01	(−0.04, 0.03)	**0.44**	(0.35, 0.54)
Dose	**−0.10**	(−0.19, -0.01)	−0.01	(−0.04, 0.01)	**−0.11**	(−0.21, −0.02)
Expectations – 6 weeks (R^2^ = 0.40)						
Expectations – baseline	**0.15**	(0.06, 0.25)	0.05	(−0.01, 0.12)	**0.21**	(0.09, 0.32)
Doctor-Patient encounter – 6 weeks	**0.20**	(0.11, 0.30)	**0.12**	(0.07, −0.17)	**0.32**	(0.21, 0.43)
Low back pain – 6 weeks	**−0.54**	(−0.63, −0.45)			**−0.54**	(−0.63, −0.45)
Low back pain – baseline	**0.20**	(0.11, 0.30)	**−0.20**	(−0.28, −0.11)	0.01	(−0.09, 0.11)
Dose	**0.15**	(0.07, 0.23)	**0.07**	(0.01, 0.13)	**0.22**	(0.13, 0.32)
Doctor-Patient encounter – 12 weeks (R^2^ = 0.37)						
Expectations – baseline			**0.11**	(0.04, 0.18)	**0.11**	(0.04, 0.18)
Doctor-Patient encounter – 6 weeks	**0.55**	(0.45, 0.65)	**0.05**	(0.04, 0.18)	**0.59**	(0.49, 0.69)
Low back pain – 6 weeks	−0.01	(−0.13, 0.10)	**−0.07**	(−0.08, −0.06)	−0.09	(−0.20, 0.03)
Expectations – 6 weeks	**0.13**	(0.01, 0.25)			**0.13**	(0.01, 0.25)
Low back pain- baseline			0.00	(−0.08, 0.08)	0.00	(−0.08, 0.08)
Dose	−0.07	(−0.15, 0.02)	0.06	(0.00, 0.13)	−0.01	(−0.11, 0.09)
Low back pain – 12 weeks (R^2^ = 0.54)						
Expectations – baseline			−0.05	(−0.12, 0.02)	−0.05	(−0.12, 0.02
Doctor-Patient Encounter – 6 weeks	0.06	(−0.03, 0.14)	**−0.20**	(−0.26, −0.14)	**−0.15**	(−0.25, -0.04)
Low back pain – 6 weeks	**0.59**	(0.49, 0.69)	**0.07**	(0.06, 0.08)	**0.66**	(0.55, 0.76)
Expectations – 6 weeks	**−0.12**	(−0.21, -0.03)	**−0.01**	(−0.02, −0.00)	**−0.13**	(−0.22, −0.04)
Doctor-Patient encounter – 12 weeks	−0.06	(−0.15, 0.03)			−0.06	(−0.15, 0.03)
Low back pain- baseline	**0.15**	(0.06, 0.24)	**0.26**	(0.19, 0.33)	**0.41**	(0.31, 0.51)
Dose	−0.03	(−0.10, 0.03	**−0.09**	(−0.15,−0.02)	**−0.12**	(−0.21, -0.03)
Expectations – 12 weeks (R^2^ = 0.58)						
Expectations – baseline	0.03	(−0.05, 0.10)	**0.15**	(0.07, 0.23)	**0.18**	(0.05, 0.30)
Doctor-Patient encounter – 6 weeks	−0.04	(−0.13, 0.06)	**0.30**	(0.22, 0.38)	**0.26**	(0.13, 0.40)
Low back pain – 6 weeks	0.04	(−0.08, 0.16)	**−0.49**	(−0.56, -0.43)	**−0.45**	(−0.58, −0.32)
Expectations – 6 weeks	**0.63**	(0.53, 0.73)	**0.04**	(0.02, 0.07)	**0.68**	(0.57, 0.78)
Doctor-Patient encounter – 12 weeks	**0.13**	(0.05, 0.21)	0.01	(−0.01, 0.03)	**0.14**	(0.06, 0.23)
Low back pain – 12 weeks	**−0.22**	(−0.32, −0.12)			**−0.22**	(−0.32, −0.12)
Low back pain- baseline			−0.06	(−0.14, 0.03)	−0.06	(−0.14, 0.03)
Dose			**0.16**	(0.08, 0.23)	**0.16**	(0.08, 0.23)

*Standardized coefficients (β) and 95% confidence intervals (95% CI) are presented for direct effects (path coefficients connecting two variables in Figure 2), indirect effects (computed from all multi-arrow paths connecting two variables), and total effects (sum of direct and indirect effects). Bolded coefficients are statistically significant (p < .05).

R^2^ shows the proportion of the total variation of each dependent variable that is explained by the model. R^2^ = 0.36 for the entire model.

Main findings: The principal finding was that the DPE evaluated at the end of care and SMT dose had similar effects on pain outcomes. DPE β = −0.22 and −0.15 and dose β = −0.11 and −0.12 for the six and 12-wk pain outcomes, respectively. The unstandardized dose effect on 12-wk pain (using the original 100-point pain scale) was −2.2 points per 6 visits. The negative signs indicate that a more favorable view of the DPE and a larger dose of SMT were associated with less pain at follow-up. The other major finding was that baseline expectations had little total effect on pain for both follow-up time points (β’s = −0.05, p > .05).

Low back pain: LBP intensity at a given time point was the strongest determinant of future LBP intensity. For example, 6-wk and baseline pain were the greatest determinants of 12-wk pain (β = 0.66 and 0.41, respectively). Six-wk LBP had notable effects on follow-up expectations (β = −0.54 and −0.45). The negative signs show that greater pain was associated with poorer confidence that treatment was working after completion of care and later follow-up. Conversely, at baseline greater pain was associated with greater confidence in the success of any study care (β = 0.27).

Dose: As shown above, there was a small linear dose effect on pain at the end of care and at the primary follow-up time point. A greater number of SMT sessions also produced greater confidence that the treatment is working. However, dose had no notable influence on the DPE for both follow-up time points (|β| ≤ 0.05, p > .05).

Doctor-patient encounter: The DPE evaluated at the end of care was a strong determinant of the DPE score at 12 weeks (β = 0.59). and, as mentioned above, was a determinant of low back pain at both follow-ups. More favorable perception of the DPE at the end of care was associated with greater confidence that treatment was working at both follow-ups (β = 0.32 and 0.26). As mentioned above, the DPE was a determinant of low back pain at both follow-ups. When a sensitivity analysis was conducted with reverse pain-DPE paths in the model, the path coefficients were trivial indicating that pain was a poor determinant of DPE.

Expectations: Expectations affected future expectations. Notably, confidence that care is working at 6 weeks was strongly related to confidence at 12 weeks (β = 0.68). Greater expectations were associated with more positive future DPE and better pain outcomes at the 12-wk follow-up.

### Effects of SMT dose groups

When the linear dose variable was replaced in the secondary analysis with three indicator variables comparing each treatment group to the control, all non-dose path coefficients and effects were left essentially unchanged. The results for dose paralleled those presented above with the largest effects found for the highest two dose groups, particularly for 12 sessions of SMT. There were only two notable effects of dose on pain intensity, namely for 12 SMT sessions of SMT at both follow-ups (β = −0.18). This corresponds to an advantage for 12 SMT sessions over the control of −7.1 and −8.5 points on the 100-point scale, respectively.

## Discussion

### Doctor-patient encounter

Our study underscores the importance of controlling the encounter between patient and provider in unblinded efficacy studies. The DPE can make a relatively important impact on outcomes, and differential behavior on the part of study treatment providers may have a confounding effect. We recommend that investigators report on method and degree of success in controlling the DPE.

This study confirms that chiropractors practicing in private clinics can maintain equipoise in providing study care in a randomized controlled trial with minimal training and oversight. The treating chiropractors were instructed to “be themselves” in their interaction with the participants and demonstrate equal enthusiasm across all study groups. Table 1 shows that in addition to success in achieving uniformity in participant perception of clinician enthusiasm, the study achieved balance in perception of clinician comfort with and confidence in care. Equipoise was demonstrated for type of intervention (spinal manipulation or brief light massage) and dose (number of visits for interventions) in this study of LBP and our previous study of cervicogenic headache [14].

It is well known that the DPE can have notable health benefits [23]. This study showed the relative effect of the DPE on efficacy under the conditions of a randomized trial, where treatment and what clinicians can say to the participants are restricted by study protocol. Practitioners should consider ways to benefit the patient by enhancing this effect in general practice.

### Expectations

Baseline expectations in this study influenced pain outcomes neither directly nor indirectly through its effects on later DPE and confidence in care. The mixed results found in the literature [5-9] for the association of baseline expectations with outcomes in LBP studies may be attributable to differences in expectation instrument, outcome measures [6], treatment and control interventions, magnitude of treatment effect, patient population, study design (placebo-controlled or comparative study), timing relative to informed consent and randomization, modeling techniques (e.g., regression or causal modeling), or variables included in the model.

Controlling baseline expectations in the conduct and analysis of randomized trials can also be challenging. Baseline expectations for each intervention can be balanced across groups through randomization. However, expectations in the participant population can be different for each study intervention, and hence, the mean expectation for participants’ assigned treatment can be different across treatment arms [14]. The analysis could include adjustment for patient expectation for any or all of the treatments or just the expectation for the assigned treatment. The choice of expectation covariates can yield different adjusted treatment effects. Disclosure rules about placebo and sham interventions can also affect baseline expectations, an additional reason to include expectations in the analysis of treatment effects.

Expectations of treatment success reported at the end of care and at later follow-up were not equal across groups; the higher dose groups had greater confidence treatment was working (Table 1). This may be attributed to the fact that pain had a strong effect on expectations and higher SMT dose groups had better pain outcomes. Coupled with this, higher dose was also associated with greater expectations. This illustrates how expectations after baseline might more appropriately be considered an outcome rather than a nuisance variable that can be adjusted for in the analysis.

### Model effects

This study demonstrates the importance of examining indirect and total effects in addition to the direct effects or path coefficients in Figure 2 and Table 3. Indirect effects can significantly enhance or cancel direct effects, so exclusion of them can be misleading. For example, the path coefficient for the effect of dose on 12-wk LBP (β_direct_ = −0.03) could lead to the conclusion that the quantity of SMT visits had no causal relationship with LBP at this time point. However, inclusion of the indirect effect of dose on 12-wk pain, mediated predominantly through 6-wk pain (β_indirect_ = −0.09), yielded a statistically significant total effect at 12 weeks (β = −0.12). This is comparable to the causal relationship with pain at 6 weeks (β = −0.11). A canceling influence of an indirect effect is illustrated by the relationship of baseline LBP to 6-wk expectations. The direct effect was β_direct_ = 0.20, although the near null total effect demonstrated the absence of a relationship (β = 0.01).

A comparison with our previous path analysis model for cervicogenic headache is difficult because sample size of that preliminary model limited the number of paths that could be included and the precision of path coefficient estimates [14]. A major similarity between the two studies was the finding of an effect of pain on expectations. The most important difference between the two was that relative to the effects of DPE and expectations, there was a much larger effect of treatment on pain in the headache study.

### Limitations

There are two principal limitations of the study. The first is the limited number of variables used to quantify the DPE. Our purpose was to observe the differential effects of patient perception of the doctor on patient outcomes in an open-label randomized trial. More extensive evaluation with a fully validated instrument might paint a different picture of DPE impact. The second major limitation is the structure of the model itself. Both the variables included and the paths of influence selected can affect path coefficients [32,33]. In our case, we are encouraged by the fact that the differences between SMT dose groups and control group presented in the secondary analysis above are virtually identical to the findings of the primary analysis for this randomized trial [15]. A final caution must be paid to the generalizability of DPE findings from a randomized trial to the clinical setting. The DPE here was carefully circumscribed by the protocol, particularly when it came to giving patients advice. In clinical practice the patient-provider interaction is potentially much broader and much richer in character.

## Conclusions

The doctor-patient encounter can have a relatively important effect on outcomes in open-label randomized trials of treatment efficacy, both directly and mediated indirectly through participant expectations. Therefore, attempts should be made to engender equivalence of the patient perception of the encounter across treatment groups and then report on the success of that effort. Even with limited training of the treating chiropractors in this randomized trial, we were successful in achieving uniformity in overall patient perception of the doctor’s enthusiasm, comfort with treatment, and confidence across groups.

## Abbreviations

LBP: Low back pain; SMT: Spinal manipulative therapy (spinal manipulation); DPE: Doctor-patient encounter; β: Standardized coefficient for the effect of care (direct, indirect, or total).

## Competing interests

The authors declare that they have no competing interests.

## Authors’ contributions

MH and DV made substantial contributions to conception, design, funding acquisition, and supervision of the randomized trial and creating the path analysis models. MBN and NP made substantial contributions to model design; they conducted the analysis. All authors contributed to interpretation of data and manuscript preparation. All authors approved the final manuscript.

## Pre-publication history

The pre-publication history for this paper can be accessed here:

http://www.biomedcentral.com/1472-6882/14/16/prepub
